# Challenges facing COVID-19 vaccination in India: Lessons from the initial vaccine rollout

**DOI:** 10.7189/jogh.11.03083

**Published:** 2021-06-26

**Authors:** Abhishek Pandey, Pratha Sah, Seyed M Moghadas, Sandip Mandal, Sandip Banerjee, Peter J Hotez, Alison P Galvani

**Affiliations:** 1 Center for Infectious Disease Modeling and Analysis (CIDMA), Yale School of Public Health, New Haven, Connecticut, USA; 2 Agent-Based Modelling Laboratory, York University, Toronto, Ontario, Canada; 3 Indian Council of Medical Research, New Delhi, India; 4 Department of Mathematics, Indian Institute of Technology Roorkee, Uttarakhand, India; 5 National School of Tropical Medicine, Baylor College of Medicine, Houston, Texas, USA

With more than 28 million reported cases as of June 5, 2021, India continues to be one of the countries worst hit by the COVID-19 pandemic. After months of steady decline in cases since September 2020, India is now battling a devastating second wave, reaching a global record of more than 400 000 reported cases in a single day at the peak. The resurgence coincides with the emergence of the Delta variant that may be more transmissible [1]. In an attempt to control the COVID-19 pandemic, India initially authorized the emergency use of two vaccines, each requiring two doses – Covishield developed by Oxford/AstraZeneca and Covaxin developed by Bharat Biotech in collaboration with Indian Council for Medical Research (ICMR) and the National Institute of Virology The vaccination drive in India started on January 16, 2021 with an ultimate target of vaccinating 300 million people by August 2021 [2]. The first phase of vaccine roll-out prioritized 30 million health care and frontline workers. Vaccine rollout however has been slower than expected and the country is now facing shortages due to an inadequate scale-up of vaccine production so far. Here we discuss the challenges facing COVID-19 vaccination in India using state-level vaccination data during the initial stage of vaccine rollout.

**Figure 1:**
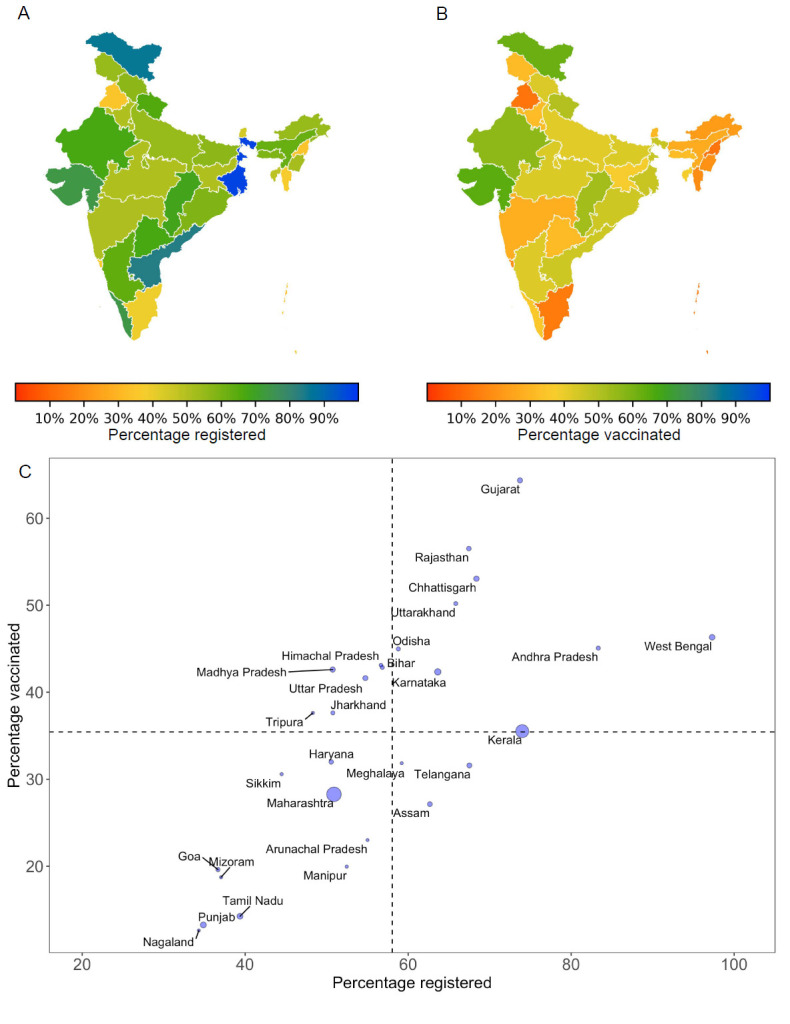
Phase 1 vaccine rollout in India. Percentage of eligible individuals that (**A**) registered for vaccination and (**B**) received at least the first dose of vaccine by February 28, 2021. We chose February 28, 2021 as the end time point because Phase II vaccinations started on March 1, 2021. We calculated the state-level number of eligible individuals by using data on the number of health care and frontline workers [3-5], and normalizing the total eligible across states so that the national total added up to the target of 30 million. (**C**) Comparison of percentage registered and vaccinated across states. Dashed lines indicate national averages – 35.44% vaccinated (horizontal dashed line) and 58.06% registered (vertical dashed line) of those eligible for Phase I vaccination by February 28, 2021. Point size is proportional to the number of active COVID-19 cases in the state on February 27, 2021.

Out of the 30 million individuals prioritized for the first phase, 18 million registered for vaccination and 11.1 million received their first dose. Only 2.46 million people received the second vaccine dose, translating to an uptake rate of 8% nationally. As public health management in India is decentralized, the state governments are primarily responsible for vaccine distribution. We calculated the rate of vaccine uptake for each state by estimating the percentage of eligible individuals registered for vaccination and using data of those receiving at least the first dose of vaccine by the end of the first vaccination phase. We found stark differences in vaccine rollout across states **(****Figure 1****)**. Registration of eligible individuals ranged from over 80% in Andhra Pradesh and West Bengal to less than 40% in Nagaland, Punjab, Goa, Mizoram and Tamil Nadu. By the end of February 2021, only four states (Gujarat, Rajasthan, Chhattisgarh and Uttarakhand) were able to vaccinate at least half of those eligible with the first dose. Less than 20% of eligible individuals were vaccinated in the states of Nagaland, Punjab, Tamil Nadu, Mizoram, Goa, and Manipur. In contrast, states of Gujarat, Madhya Pradesh and Rajasthan vaccinated over 80% of those registered **(****Figure 1****,** Panel C**)**. The states of Maharashtra, Kerala, Karnataka, Punjab and Tamil Nadu accounted for more than 80% of all active cases in India by the end of February. Except for Karnataka, vaccination coverage during the initial phase was less than the national average of 35% in each of these high burden states **(****Figure 1****,** Panel C**)**.

**Figure 2:**
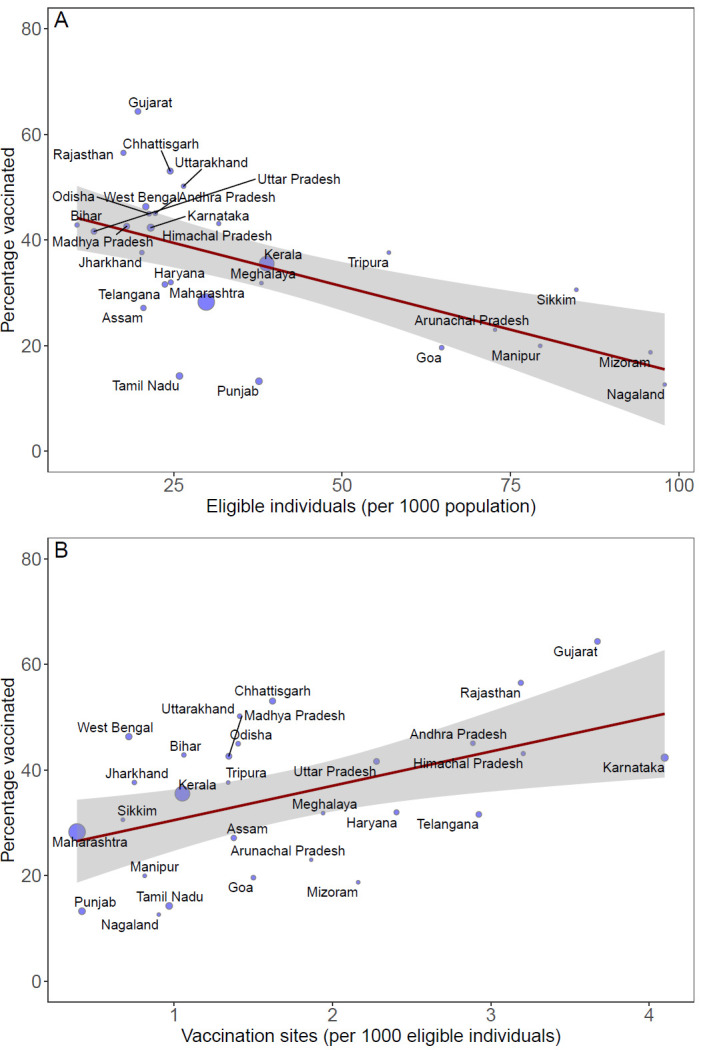
Association between percentage vaccinated with at least one dose and (**A**) number of eligible individuals per capita and (**B**) number of vaccination sites per eligible individual. The solid line is the regression line and the shaded area represents the 95% confidence interval. Point size is proportional to the number of active COVID-19 cases in the state on February 27, 2021.

To understand the heterogeneities in vaccine distribution in India, we examined the association between vaccination efforts across states with respect to the number of eligible individuals per 1000 population and vaccine delivery infrastructure. We estimated individuals eligible for the initial vaccination phase using state-level data on the number of health care workers [3], police strength [4] and prison staff [5] and normalized the data to match 30 million eligible individuals nationally. State-level vaccination data was obtained from the governments' website CoWin [6]. We found that lower vaccination coverage was associated with a higher number of individuals eligible for vaccinations per capita (**Figure 2****,** Panel A, *P* < 0.010). Northeastern states of Nagaland, Mizoram and Manipur, for instance, had more than 75 eligible individuals per 1000 population, but vaccinated less than 20% of those eligible, whereas Uttar Pradesh and Bihar with less than 20 eligible individuals per 1000 population, vaccinated more than 40% of those eligible. Vaccination uptake was positively correlated with vaccine delivery infrastructure such that the states that had a higher number of vaccination clinics per 1000 eligible individuals were able to achieve higher vaccination coverage by the end of the initial phase of vaccination (**Figure 2****,** Panel B, *P* < 0.010). For example, Gujarat with more than 3 vaccination clinics per 1000 eligible individuals achieved a vaccination coverage of 64%; whereas with less than 1 vaccination clinic per 1000 eligible individuals, Maharashtra was only able to achieve vaccination coverage of 28%.

India grappled with low rates of both registration and vaccine administration during the initial stages of the vaccination campaign, likely because of vaccine hesitancy, misinformation propagated by social media, and technical glitches in the online registration platform. Vaccine hesitancy in India was fuelled by concerns about vaccine safety and skepticism about the efficacy of the Covaxin vaccine that was approved before the completion of the Phase III clinical trial [7]. Ensuing suspension of Covishield in multiple European and African countries due to safety concerns, together with rising anti-COVID-19 vaccine sentiments [8] may have elevated distrust. The steady decline in cases for months in India may have encouraged people to adopt a wait and watch approach, contributing to vaccine indifference. Moreover, the requirement to register online for receiving vaccines may have contributed to low vaccination rates as more than half of the Indian population does not have access to the internet. Our results showed large differences in the pace of vaccination across states likely reflecting the local budgetary constraints and disparity in health care infrastructure.

Despite the slow start, the vaccination campaign in India improved in pace as the country addressed some of the challenges. Initial glitches in the online registration platform have largely been resolved, allowing public health officials to effectively track and plan logistics for the vaccination campaign. Along with online registrations, eligible individuals aged 45 or more can now receive vaccines without pre-registration. Interim results for the Covaxin clinical trials have demonstrated a vaccine efficacy of 81% against symptomatic infections [9] alleviating concerns about its efficacy. Unlike the initial stage of the campaign, during which vaccines were available only at government clinics, states are now utilizing private hospitals to vaccinate individuals. Given that more than 80% of health care services in India are provided by the private sector [10], private hospitals can greatly accelerate vaccination rollout.

The devastating second COVID-19 wave in India has overwhelmed an already strained health care infrastructure. The country is now facing a shortage of essential medical supplies and battling a multi-pronged war to contain the second wave as well as to continue vaccination. The resurgence of cases, coinciding with the spread of highly transmissible SARS-CoV-2 variants underscores the urgency to accelerate the vaccine rollout. Although India has emerged as a COVID-19 vaccine development and manufacturing hub providing the country an edge for procurement of vaccines for domestic use, the scale of production has been inadequate. Indian vaccine manufacturers have struggled to ramp up production due to financial constraints as well as due to embargo on the export of raw materials needed for vaccine production by the United States. Extension of eligibility criteria to include all individuals above 18 years of age on May 1st would inevitably require expansion of vaccine supply and vaccination centers to accommodate the substantial increase in individuals targeted for vaccination.

To facilitate the importation of vaccines with emergency use authorization from elsewhere in the world, India has removed the requirement of a local bridging trial. The state governments and Indian corporates can now supplement their vaccine supply by importing doses directly from both domestic and international manufacturers. Efforts to procure and locally manufacture single-dose vaccines, such as those produced by Johnson & Johnson, would facilitate efforts in rapidly expanding vaccine coverage. To alleviate the financial strain and boost domestic capacity, the Government of India is making advance payments to the local manufacturers. Moreover, the United States has agreed to supply raw materials critical for scaling up production of Covishield. The United States has also decided to provide up to 60 million vaccine doses of the AstraZeneca vaccine to other countries, including India [11]. These ready-to-use vaccines can help bolster the vaccination drive in India.

**Figure:**
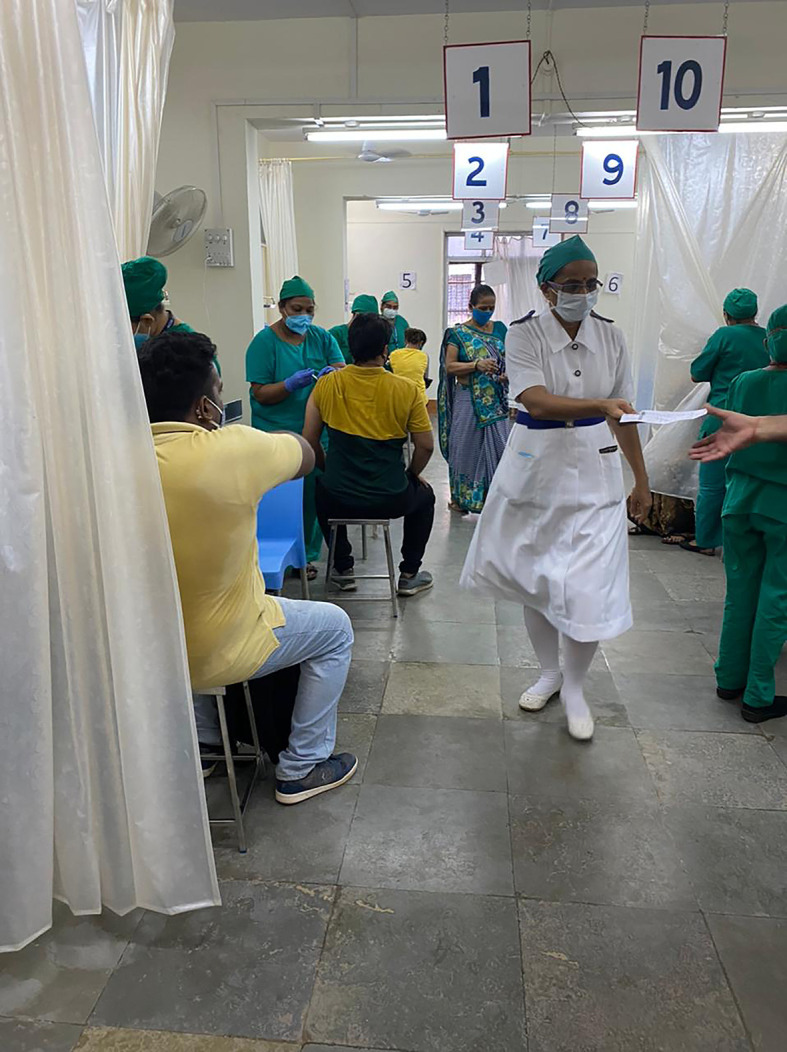
Photo: From the collection of Ashish Jain, used with permission.

The ongoing humanitarian crisis posed by the second wave of COVID-19 in India could threaten the progress made in other countries and underscores the vulnerability of countries with weak health care infrastructure. Elevated community transmission provides more opportunities for vaccine escape variants to arise, which could prolong the pandemic. Moreover, the halt of COVID-19 vaccine exports from India has impacted the already slow pace of the vaccination drives in many low-income countries. A concerted international effort is therefore imperative to assist India to curtail the transmission effectively and save millions of lives.

Achieving the ambitious target of vaccinating 300 million individuals by August 2021, while challenging, is both feasible and essential to avert an exacerbation of COVID-19 outbreak in the country. As of April 22, 2021, a total of 118. 45 million doses have been administered since the initial vaccine rollout ended, corresponding to a national average of 2.2 million doses per day, which is over 6 times higher than the average daily vaccination rate of 325 000 during the initial phase [12]. On April 22, 2.7 million doses were administered. At this rate, the goal of vaccinating 300 million people could only be achieved by early October 2021. If the vaccination rate is increased to 3.37 million doses per day, the goal could be reached by August 2021. Going forward, India’s ability to achieve its objectives of vaccination campaigns will depend on boosting vaccine supply, expanding health care capacity, staffing more health care professionals to administer COVID-19 vaccines, overcoming vaccine hesitancy and misinformation, and ensuring an equitable distribution of vaccines.
